# Slaughtering for a living: A hermeneutic phenomenological perspective on the well-being of slaughterhouse employees

**DOI:** 10.3402/qhw.v11.30266

**Published:** 2016-04-20

**Authors:** Karen Victor, Antoni Barnard

**Affiliations:** 1 HR Manager at Dawn HR Solutions Pty Ltd, Alberton, South Africa; 2 Department of Industrial and Organisational Psychology, University of South Africa, Pretoria, South Africa

**Keywords:** slaughterhouse work, slaughterfloor employee, hermeneutic phenomenology, life-world research, post-traumatic stress syndrome, PTSD

## Abstract

Slaughterhouses constitute a unique work setting exposing employees to particular physical and psychological health challenges. Research that focuses on the well-being of slaughterhouse employees is limited, and the aim of this study was to explore their well-being by conducting a hermeneutic phenomenological study of specifically the slaughterfloor employees’ work-life experiences. The study was conducted in a South African commercial abattoir setting. Thirteen slaughterfloor employees and two managers of the slaughterfloor section participated in unstructured interviews. A hermeneutic phenomenological approach to data analysis was adopted following the stages of a naïve reading, a structural thematic analysis, and a comprehensive understanding. Data analysis resulted in four process-related themes representing the different stages of becoming a slaughterer, (mal)adjusting to slaughter work, coping with and maintaining the work, and living with the psycho-social consequences of slaughter work. Results facilitate an understanding of how employee well-being manifests in each of these stages of being a slaughterfloor employee. The risk potential of employees suffering from post-traumatic stress syndrome was evident throughout the stages of being a slaughterfloor employee and offers a useful diagnostic framework to facilitate employee well-being assistance. Slaughterhouse management should develop a holistic focus addressing employee well-being needs evident in each of the stages of being a slaughter worker and by extending well-being interventions to the broader communities that the slaughterhouse functions in.

Workplace factors and the psychological well-being of employees are inextricably linked. Work conditions, job demands, and the availability of resources significantly affect employees’ general and work-related coping and psycho-social adjustment (Lowman, 1996; Schaufeli, Bakker, & Van Rhenen, 2009). Undesirable work conditions, such as limited autonomy, high psychological and physical demands, extended working hours, job insecurity, workplace violence, injury and discrimination, have particularly been shown to cause poor psychological health (Hillier, Fewell, Cann, & Shephard, 2005; Steyn & Vawda, 2014). Some occupations characteristically imply a higher exposure to trauma, violence, and stress-related contexts, which holds a greater risk potential to employees’ well-being (Steyn & Vawda, 2014; Young, Koortzen, & Oosthuizen, 2012). In particular, slaughterhouse employees have been singled out as high-risk cases for adverse health effects (Dillard, 2008; Fitzgerald, 2010; MacNair, 1999; Van Holland, Soer, De Boer, Reneman, & Brouwer, 2015).

Slaughterhouse work is characterised by high staff turnover, absenteeism, and disciplinary actions (Broadway, 2007; Dalla, Ellis, & Cramer, 2005). This is due to the physically demanding and often monotonous nature of the work (Van Holland et al., 2015). Workers have to contend with an inherently hazardous work context since they handle dangerous cutting tools at extreme production speeds (Fitzgerald, 2010; Human Rights Watch, 2004). Slaughterhouses have some of the highest reported injury rates in the manufacturing industry (Beirne, 2004; Broadway & Stull, 2006). Injury rates have been reported to be as high as 20–36% per annum (Dalla et al., 2005; Dillard, 2008; Human Rights Watch, 2004; Olsson, 2002). The physically demanding nature of the work is exacerbated by the long shifts in a damp and cold environment (Human Rights Watch, 2004). Monotonous and repetitive movements in the production line coupled with a consistent urge to speed up production, cause employees to suffer from various ailments. These include carpal tunnel syndrome, “trigger finger,” back problems, and tendonitis (Pearson, 2004). These employees also suffer sprains, cuts, punctures, back pain (Broadway & Stull, 2006), “white finger” and cut wounds (Dillard, 2008), as well as musculoskeletal disorders, “claw hand,” ganglionic cysts, bursitis, and arthritis (Human Rights Watch, 2004).

Apart from the physically dangerous employment conditions, the underlying violent nature of working in a slaughterhouse (Barmak, 2010; Broadway & Stull, 2006; Olsson, 2002) also poses a risk to the psychological well-being of employees and cases of cumulative trauma disorder have been reported (Dalla et al., 2005; Kristensen, 1991). Slaughterhouse employees, furthermore, often lack adequate resources to cope with the strenuous environment. This is mostly due to their poor socio-economic background, lack of training, and the shortage of safety equipment at the site (Fitzgerald, 2010; Human Rights Watch, 2004). In addition, violence against animals has been linked to psychological health problems in humans (Beirne, 2004; Daly & Morton, 2008; Henry, 2004; Porcher, 2011). Consequently, deviant behaviour patterns of slaughterhouse employees have been reported in and outside of the work setting (Fitzgerald, Kalof, & Dietz, 2009) with specific reference to social dilemmas such as substance abuse, intimate partner violence, and an increase in crime rates (Fitzgerald, 2010).

In summary, the slaughterhouse presents a work context with an undercurrent of violence, persistent trauma, stringent and monotonous production routines, health hazards, and physical strain (Van Holland et al., 2015). In the South African context, these working conditions are coupled with the previously mentioned fact that employees originate from the lower socio-economic spectrum of society. Having only basic education and training and being faced with the reality of low income and limited family resources, slaughterhouse employees seem particularly taxed in their capacity to maintain their psychological health.

South African research on slaughterhouses focuses predominantly on the impact various factors have on the quality and cost-efficient production of meat (Chulayo, Tada, & Muchenj, 2012; Vimiso & Muchenj, 2013). In general, predominant issues of concern include hygiene within the meat-processing environment, animal's living conditions, and pre-slaughter stress (Porcher, 2011). Research focusing on the well-being of slaughterhouse employees is scarce and in the South African context almost non-existing. The problem underlying this study is a lack of understanding on work-life experiences within the slaughtering environment, and its consequences for employee well-being. The aim of this study was, therefore, to obtain a critical understanding of the well-being of slaughterhouse employees working in the slaughterfloor section of a commercial South African abattoir.

## Method

We approached this study from a hermeneutic phenomenological stance assuming a constructivist epistemology and perspectival reality when exploring well-being as a phenomenon in the work-life experiences of slaughterhouse employees. Evolving from the pure phenomenological description of meaning and experience, hermeneutic phenomenology accounts for the belief that a critical interpretation of phenomenological experience is not only inevitable but also required in rigorous scientific research (Kafle, 2011). Meaning is co-constructed through a critical interpretation of participants’ narrative experiences and the integration of the researcher's theoretical and experiential preconceptions into the research findings (Laverty, 2003; Norlyk & Harder, 2010). The primary researcher was employed in the human resources section of the abattoir and her preconceptions were much influenced by her own shocked response and abhorrence when observing the slaughterfloor for the first time. From her personal reflective notes, she recalls “The moment was horrible and emotionally disturbing to me. I questioned the fact that people love to eat meat. I struggled to reconcile myself with such brutality and the traumatic effect stayed with me for days.” This experience and her continued work with employees’ disciplinary hearings and grievances sparked a growing concern for the employees’ psychological well-being and pre-empted the initial idea for the study. The secondary researcher, an academic and psychologist registered with the Health Professions Council of South Africa (HPCSA), was intrigued by the predicament of the slaughterfloor employees who also clearly represented the lower socio-economic spheres of the South African society. Our preconceptions were therefore specifically directed from a pragmatic and personal perspective as well as from a scientific interest in exploring and understanding coping and adjustment in the work context. Commencing with the study, we found much evidence in the literature of the physical and psychological impact of slaughterwork in studies such as Fitzgerald et al. (2009) and Beirne (2004), yet no South African research in this regard.

Following the ideographic nature of hermeneutic phenomenology, we employed methods typical to reflective life-world research (see Dahlberg, Dahlberg, & Nyström, 2008) which become clear in the following discussion.

### Research context and participants

This study was conducted in the slaughterfloor section of a large commercial, Halaal-certified South African abattoir with the capacity to process up to 2000 cattle per day. The participants to the study included employees working specifically on the slaughterfloor. These employees are responsible for the actual killing in the production process. This slaughtering entails stunning the animal unconscious with a captive bolt on the head and then killing the unconscious animal by cutting its jugular vein. Fifteen slaughterfloor employees were employed on a shift-work basis at the abattoir during the time of the study.

Purposive, non-random, and convenience sampling was applied and ultimately 14 of the 15 slaughterfloor employees participated in the study. Two of the fourteen participants occupied supervisory positions on the slaughterfloor. All of the participants were men. Six participants were Muslim, whereas the religion of the other participants was not made known. Five participants were younger than 30 years, with seven between the ages of 30 and 40 years. One participant fell into the 31 to 40 age group and one was older than 60 years. The sample included nine Africans, one Indian, and four Caucasians. All participants had a schooling level that ranged between Grade 10 and 12.

### Data collection

We conducted unstructured interviews typical to life-world research (Dahlberg et al., 2008) and for eliciting life stories (Van Manen, 1997). Thus, following the advice on unstructured interviewing by Dahlberg et al. (2008, p.190), we prepared a general opening question and a “few areas of interest” to be used as directive questions when needed. After the opening question, we allowed the discussion to develop naturally through probing and follow-up questions. A general opening question “Tell me about your work and what you do every day” initiated the interviews with slaughterfloor employees. Congruent to Van Manen's (1997) interview approach in researching lived experience, our aim was to gather narrative data as a rich resource for exploring the well-being of slaughterfloor employees. The unstructured approach we followed therefore also reflected elements of the narrative interview as described by Lindseth and Norberg (2004) as probing questions directing the participants to narrate examples of their good and bad job-related experiences, when and how they started to slaughter, how they were coping with work-related difficulties, and how their jobs impacted their personal lives and relationships. We attempted not to ask leading questions as the focus was rather that the uncovered issues relating to well-being should emerge naturally from the participants’ work-life stories.

The duration of the initial interviews ranged between 40 and 60 min. Although English was not the home language of any of the participants, they all had the ability to converse in English. Only one interview required the use of a translator to ease communication flow and understanding. Two follow-up interviews were conducted of approximately 20–30 min. One participant requested a follow-up interview because he felt that he still wanted to discuss his work, another interview was interrupted with a work-related crisis, and a follow-up interview was scheduled to continue and finalise the discussion. Recorded interviews were transcribed and documented in MSExcel for data analysis.

### Data analysis

The analysis of the collected data followed a hermeneutic phenomenological approach and we were guided by the analytic processes described in Dahlberg et al. (2008) and Lindseth and Norberg (2004). In our initial immersion into the data, we adopted a naïve reading of the text to obtain a holistic sense of meaning and perspective on well-being in the slaughterfloor working context by re-reading the text several times. Next, sections of meaningful texts were condensed and given a descriptive understanding. With the naïve reading and research aim consistently in mind, the sections of meaningful texts and their condensed meaning were revisited and interpreted to form sub-themes and themes, presenting our structural thematic analysis.

Our initial interpretations resulted in four process-related themes. These themes were clustered and finally investigated closely to establish their relevance and relationship to one another and to our overall understanding of the text. To verify the data throughout the analytic process, we moved from the whole to the different parts of meaning, back to the whole, in an iterative manner. Thus, we consistently verified our structural thematic analysis against the aim and naïve reading and our interpretation of the whole ultimately delivered a comprehensive understanding of slaughterfloor employees’ well-being. Finally, trustworthiness was sought by critically reflecting on relevant theory and our own preconceptions and evolving knowledge, against the comprehensive understanding, the structural thematic analysis, and the naïve reading in the context of the research objective.

### Ethics

Access was gained to the organisation and the slaughterfloor employees since the primary researcher is employed in the human resources section of the abattoir. Written permission was first obtained from the abattoir's management to conduct the study. During a meeting with the slaughterfloor section employees, the primary researcher explained the research project in terms of its aim, namely wanting to gain a better understanding of slaughterers’ well-being by discussing with them their work and life experiences. They were told who would be involved in the project and how confidentiality and anonymity would be ensured. The primary researcher then arranged with them that she would contact them each privately to request their participation. Employees were thereafter met with individually and the nature and aim of the study as well as who would be involved and how their anonymity would be protected and confidentiality ensured was again clarified. The freedom to participate and also to withdraw at any stage, as well as the need to record the interviews for further analysis, was also explained at this stage. Interviews were then scheduled if the employee consented to participate. Two employees chose not to participate. The aim of the study and anonymity and confidentiality issues were again reiterated at the start of each interview as well as in the two follow-up interviews. Participants were assured that their identity would not be disclosed in any way and their permission to record the interview was sought. One participant requested a translator and the translator was requested to sign a confidentiality agreement after being briefed about the ethical requirements. Interviews were conducted in a private office away from the production building at the abattoir during normal working hours. This research was directed by the ethics policy of UNISA (UNISA, 2013) as well as the ethical code of the HPCSA (Department of Health, 2006) regulating the psychology profession in South Africa.

## Findings

We do not claim objectivity in the reporting of the results but present a co-constructed understanding of well-being in the slaughtering context. Our combined experience of the slaughterfloor and a theoretical pre-understanding of employee adjustment and coping impacted our interpretations. Following the naïve reading, the structural thematic analysis is presented, wherein we attempt to remain as close to the verbatim text as possible. Then, through further iterative analysis and critical integration of our meta-theoretical predispositions and the data, we derive a comprehensive understanding of employees’ well-being in the context of slaughtering for a living.

### Naïve reading

Our naïve reading of the text impressed on us a chronology in slaughter employees’ well-being experiences from starting the job to ultimately coping and living with this unique work setting. From the data, we sensed that slaughterfloor employees go through various stages of adjusting to slaughtering animals in a work setting that is cold, bloody, and smelly. The initial experience of starting on the slaughterfloor seems to be inevitably traumatic, eliciting feelings of shock and abhorrence. Thereafter, participants report many disturbing and conflicting emotions but emphasise how these emotions change and they start to feel emotionally hardened by the work. Dreams and nightmares seem to occur frequently during the first couple of months, displaying slaughterers’ subconscious fears and anxieties. When they get more used to the work surroundings, they cope in various mental and behavioural ways, although many deny their feelings and revert to substance abuse and violence. Relying on their religious beliefs and activities as well as finding meaning in being able to provide for their families brings a sense of purpose and meaning to what they do. The consequences of working on the slaughterfloor seem to extend not only to the individual but also to their families and to the bigger community, resulting in domestic and other types of violence and poor interpersonal relations. Slaughterers deal with the fear of social rejection by sometimes withdrawing emotionally or acting out.

### Structural thematic analysis

**Figure 1:**
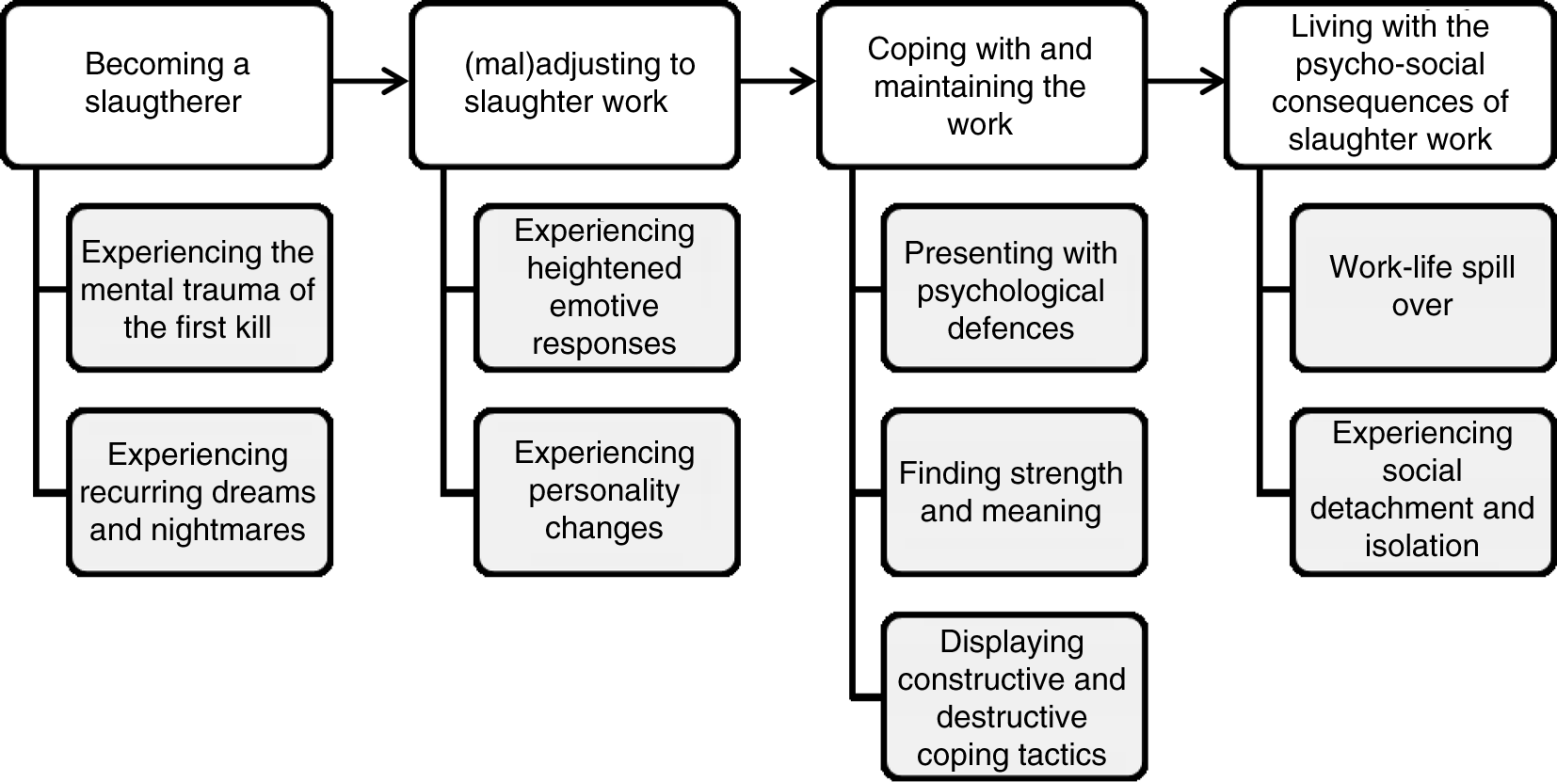
Summative presentation of themes and sub-themes.

Four themes emerged from the data reflecting our understanding of well-being in the career of a slaughterfloor employee. We captured the themes as “becoming a slaughterer,” “(mal)adjusting to slaughter work,” “coping with and maintaining the work,” and “living with the psycho-social consequences of being a slaughterer.” These themes with their sub-themes are summatively depicted in Fig. 1. Table I exemplifies the structural analysis from meaning units to sub-themes and themes.

**Table I:** Example of thematic structural analysis.

Meaning unit	Condensation	Sub-themes	Main themes
“Some of them, if you stun them they just look at you and cry… when it cries and then it gives me another thing, of eish (shivering). I like animals and now I am killing the animals. The first week before I started to stun, hey, it was difficult for me.” (RP10)	Slaughtering for the first time is very difficult	The mental trauma of the first kill	Becoming a slaughterer
“Sometimes I saw myself slaughtering the animals, but you see eyes, I saw, eyes of the animal. It's like its watching me. That thing, that dream, I didn't feel well even when I came back to work, but I keep on checking the eyes to see its watching me, because I saw it in the dream. It's not easy for a first time.” (P14)	Paranoid dreaming	Recurring dreams and nightmares	
“In my dream I see the bleeding line, just the cattle hanging on the line, all whose heads are off. I get this picture often. It's not nice to dream about blood; you wake up wet with sweat.” (RP9)	Waking up with fear after dreaming about work		
“You rather lie than say where you work. You lie to them, you are ashamed where you work. My uncle told me you never say where you work. I was very ashamed, working with blood.” (RP4)	Feeling ashamed	Experiencing heightened emotive responses	(mal)adjusting to the slaughter work
“First, *when I see all the blood there*, when I *looking, my eyes was looking at*, aye, I don’t know what I must say, eyes was looking like red river, when *they go inside, go to by-product*. I am so scared at that time.” (RP11)	Feeling afraid		
“Maximum six months then you make a change, because if he shoots continuously it will start affecting him. He gets a murderous attitude in him. He will do it to other people. He will stab you with a knife, turn around and walk away.” (RP8)	Slaughtering changes you (more aggressive; care less about your actions)	Experiencing personality changes	
“As time passes, you get used to it. You feel nothing. You can imagine, if you kill a thing a 1000 times over and over, you wouldn't have feelings after a while. It kills you on the inside, an abattoir, it kills you. You can be full of blood, it will not bother you.” (RP8)	Emotional detachment	Psychological defences	Coping with and maintaining the work
“Ensuring that the Muslim community are consuming whatever is wholesome and lawful that makes me very happy, because I’m doing something for the community on one side.” (RP13)	Doing meaningful work	Finding strength and meaning	
“The good thing is that I'm bringing money. I'm putting food on the table.” (RP6)	To sustain a living makes it worthwhile	Engaging in constructive coping and destructive coping tactics	
“Gym helps; gholf and rugby helps me to get rid of my frustrations.” (RP9)	Leisure help to cope with frustration		
“There are guys who smoke dagga to get strength to do the job. Guys are so aggressive, every afternoon after work they go drinking.” (RP4)	Substance abuse		
“What I was having was just to hit. I need to hit, especially my girlfriend. Sometimes, even if you think you can make a mistakes you hit him because eh you don't have a heart for him. That is why most people at stunning box, they can do it, they can hit their girlfriends. Say ‘hey, I hit my girlfriend yesterday’, or ‘I beat my wife yesterday’. That things we do it.” (RP5)	Violence from the work context spills over to personal life	Work-life spill-*over*	Living with the psycho-social consequence of slaughter work
“Some of my friends rejected me after working here. They said I'm a killer, I can't go with them. They say I have a gun and they don't trust me anymore. They were scared of me. They heard me shouting while dreaming about cattle.” (RP10)	Experiencing social rejection from friends	Experiencing social detachment and isolation	

### Becoming a slaughterer

*The mental trauma of the first kill*. Slaughter employees inevitably remember their first encounter with the slaughterfloor and having to slaughter. They recall vivid images of blood and describe the experience as traumatic, feeling overwhelmed by the immediate requirement to kill and the anticipation of having to slaughter hundreds of animals on the very first day. During their first kill, slaughter workers remember feeling upset and experiencing physical shock manifested by shaking and shivering. RP8 recalls: “When I shot my first animal, I started shaking” and RP10: “I was so scared, even when holding the gun, I was shaking.” Slaughterfloor employees were also emotionally disturbed by their first-time kill and noted feeling pained, saddened, and shameful. In the words of RP9 reflecting on his first slaughter, the traumatic experience of the first kill is evident as well as how this emotive experience fades into detachment which is discussed as part of the (mal)adjustment phase below:

The first time when I killed it was not easy for me. I feel pity for it. I felt I just wanted to close my eyes, turn around, and run away. It was really sad but the more you do it the easier it gets. Like yesterday I had to shoot cows in the kraal. I climbed over the fence, walked to the cow, and just shot it. I feel nothing anymore. In the beginning it was very bad.

*Recurring nightmares and dreams*. During their initial employment phase and in the immediate couple of months thereafter, slaughterers frequently have vivid dreams about their work. Slaughter employees narrated paranoid nightmares and dreams filled with fear and anxiety. In fleeing from vengeful cattle, being confronted by slaughtered cattle who fail to die, seeing animals in pain, fighting with and being watched by animals, feelings of guilt, shame, and fear are reflected in their dreams:

One day I dream that the cow gets out at the stunning box. It was alive. Then, I think that I am crying and running, and that time I am not running. Down here! Down here! [motioning that he fell down]. The cow is coming and you fall down! You fall down! (RP3)

I dream about the cattle, when you stun it, it just fall down, after falling down, when you open the door it will ask you: “Why are you killing me?” (RP10)

### (Mal)adjusting to slaughterwork

*Experiencing heightened emotive responses*. During the initial phases of employment, slaughterers experience a range of heightened negative emotions. Fear and anger seem paramount in their emotive responses to slaughtering: “I lose it quickly, I don't know why, if someone just messes with me a little bit, then yes the fists swing” (RP9). From fear and anger, feelings of shame and guilt also surface: “From the first day the shame you get from the first day, the shame you get it from starting to work here, but after that it just goes like that. Just like that” (RP5). Slaughter employees convey a moral concern believing that they will have to answer for their slaughtering actions when they die. Feelings of sadness were also frequently mentioned in slaughter stories:

They said eh, today we want you to slaughter so this amount of animals, when you look at this animals, they are so big, you say sjoh, it's me who are supposed to slaughter all these, sjoh, because you are one, all these animals have to pass through you. It's a lot of pressure and you feel depressed, because of those animals, you have to just pass here on you each and every animal. (RP12)

Lower frustration tolerance and heightened levels of irritation are experienced. RP8 narrates how previously normal issues at home, such as the house pets, now easily irritates him: “I can kick it if I want to because I kill cattle every day. Kick this dog or cat so that it flies just because you can, you don't worry see it feels like I must hurt this other animal.”

*Experiencing personality changes*. Slaughter workers emphasise the change they experience personally and also detect in their co-workers after having worked on the slaughterfloor for a period of time. Participants report that slaughtering affects their ability to think clearly and they note feeling “mad” (RP5). Doing slaughter work also affects them in a way that they seem to become more aggressive than before with a concomitant careless attitude about the consequences of their actions on other people. Slaughterers feeling emotionally much less affected is reflected in RP13's story:

So, it is really, even the first day when I came to this company it affected me, I had nightmares about the slaughtering. I have never been in an environment of blood and, but slowly, but slowly, before if I see somebody injured, or an accident, even up to today now in my life, if I see an accident that happens on the road, I will not be able to walk and look at it, I have such a, I don't know, I'm very weak, I cannot go and look at the accident, how the accident happened. On this side of the slaughtering, the first, one month in the slaughterfloor, it was very difficult, but today I'm very used to it. I even play with the blood with my boot, moving around, it's really changing people.

The heightened emotions of fear, anxiety, guilt, shame, and sadness seem to subside as time progresses on the slaughterfloor. These emotions seem to change and become less extensive—fear fades and feelings of pity and guilt become of less consequence. The range of negative emotions initially experienced and the reported emotive change during the adjustment phase made us aware of the potential risk of maladjustment and links to the coping resources they start to employ in order to maintain their capacity to work.

### Coping with and maintaining the work

*Psychological defences*. In their attempt to cope with the work and maintain their productivity, slaughterfloor employees exhibit certain psychological defences and coping mechanisms. Psychological defences such as emotional detachment, and feeling invincible, seem to undermine these slaughterers’ ability to cope. Fear and anger coupled with guilt and shame (from the adjustment phase) seem to evolve into a response of emotional detachment. The incident that RP9 witnessed demonstrates the emotionally blunting effect of the violence: “We had an incident last night; someone was cut in the stunning area, by one of his friends. He didn't even say sorry. He said it was an accident, turned around and continued cutting open the cattle.”

One respondent (RP8) explained that he copes with the effect of slaughtering by separating his work self from his personal self. Thereby, he maintains separate personality and behavioural characteristics in and outside the work environment: “You can divide yourself into two lives. Inside you are like this and outside you are different.” In the context of slaughterhouse work, Dillard (2008) refers to the separating of the self to cope with trauma as indicative of emotional suppression. He identifies this psychological coping response as “doubling.”

A number of other participants reported feelings of being invincible and superior, capable of anything, fearless, and untouchable. RP9 stresses: “You feel like you can do things that other people can't do.” Participants assert that slaughter work gives them a sense of power and causes others to fear them. From his side, RP10 boasted: “I am not afraid anymore. I'm killing thousands of cattle. You won't tell me, I'm not scared of blood; I'm not afraid to slap you with my knife.”

*Finding strength and meaning*. Efficient coping is also displayed by employees with a strong religious grounding. Muslim employees, whose work identity seem to be intertwined with their strong religious beliefs, testified that they find meaning and a sense of purpose in working as a slaughterer. Such an attitude helps them cope with the adverse effects peculiar to the particular work setting. RP11 attested to finding strength through prayer: “Every day, we start to pray” and RP6 confirmed: “I stay positive, because every day I pray, I pray to God to give me strength. I read the Quran every day and I try to stay calm, not to let the job of slaughtering to change my life, you see.” Non-Muslim employees also reported that they rely on religion as a means of coping. Some of the responses attest to this fact: “The family and the church they help you” (RP2); “Before we start, I have to pray ‘Lord, help me this day so that I can work right’” (RP10); “I go to church every day, it will help me” (RP14). Slaughterers’ need to sustain a living for themselves and their families, further support a sense of meaning and purpose in persevering in the slaughterfloor work context.

*Engaging in constructive and destructive coping tactics*. Apart from practicing their religious beliefs, participants engage in constructive coping tactics by relying on family, friends (social support structures), as well as constructive leisure time and activities. Sporting activities and leisure weekends away provide distraction to cope with the traumatic and stressful work environment. Substance abuse however seem to be a prominent destructive coping tactic which participants widely and regularly engage in, in order to relax. Participants admit to regular alcohol use: “I've got a stress of the whole week, working early until late. I'm tired. Maybe if I can get a glass of alcohol to release the stress” (RP10). Regular alcohol use could potentially lead to addiction problems: “Lately I want to go after work to drink something, it feels I must. As if I want it” (RP9). Substance abuse also extends to dagga use in order to cope: “Now see if you smoke dagga it's like drinking bioplus it gives me energy. Dagga gives them a lot of energy. Yes, it makes them hard working and strong” (RP8). Ultimately slaughterers sometimes become so distressed in their need to avoid the work context that they injure themselves to be able to still get paid, yet be off sick. RP8 states: “I admit I also once cut myself on purpose with a knife, just to feel. To be honest you know the work is so hard if you cut yourself you are actually glad and you know for a month or so you will just walk around light duty.”

### Psycho-social consequences of being a slaughterer

*Job home spill-over*. The process of becoming a slaughterer, adjusting to work on the slaughterfloor, as well as coping and maintaining the work holds several psychological consequences for the well-being of the individual employee. These consequences spill-over into the individual's interpersonal relationships outside of the work setting. Due to physical exhaustion, slaughterers often go home to eat and sleep and don't have strength to attend to relational expectations. RP4 explains:

When you get home you feel tired and don't want to do anything. You don't even want to help your wife. You are tired, you worked hard. Some get divorced, others keep on struggling, others get domestic problems because of the work, you sacrifice so much, then you start neglecting your family. You get home late, your wife is angry with you, then you start complaining about your job.

More pertinently, fatigue and stress resulting from the work lead to violence and abuse at home: “I've got the short temper. When I'm alone sitting, thinking maybe if you could fight with my wife, what am I going to do about it, I'm not afraid anymore. I'm killing thousands of cattle; hey I kill 800 or 900 cattle, it's nothing that's gonna stop me to shoot only one person” (RP10). Slaughterers seem to find an expression for their anger in incidents of fighting in and outside of the work. RP8 reflects on his aggression: “You go to the shebeen, see if you can find people to beat up. It is complicated to me, it can influence you so that you start beating your wife, assault children, such things, kick animals, hurt animals. It happened to me.” Anger is often directed at defenceless others and triggered by trivial reasons with little regard to the consequences thereof: “I don't want to fight with my hands, because I will waste a lot, I will waste a lot of energy. Maybe I can take something and hit you that case closed. I will see the consequences after” (RP10).

*Experiencing social detachment and isolation*. Slaughterers feel that their families and friends disapprove and morally object to their work because it involves killing. They reflect on others’ responses to their work as including fear, shock, and distrust. Others seem to become scared of the slaughter worker, thinking them to be dangerous. For RP1, his wife's reaction is difficult to deal with: “Especially my wife, she was very scared about my job. She said, easy, this kind of job is not all right.” Family and friends respond with shock and disbelief when confronted by the nature of slaughter work and consider the job to be gruesome and unpleasant. RP9, for example, narrates that “Most can't believe that I'm doing it. It gross them out.” Such social disapproval and negative responses from family, friends, and even co-workers exacerbate the slaughterers’ feelings of shame and guilt and result in them feeling socially rejected, misunderstood, and stereotyped: “In the beginning I was ashamed to tell people where I work, especially young girlfriends. You rather lie than say where you work, because immediately they don't like you anymore. You lie to them, you are ashamed where your work. My uncle told me you never say where you work. I was very ashamed, working with blood” (RP4). As a result, slaughterers react by withdrawing emotionally from relationships or play out the stereotypes of aggression and violence. The fear of feeling socially rejected and condemned thus result in social detachment and isolation.

## Comprehensive understanding

Slaughter work is an idiosyncratic experience involving consistent exposure to work circumstances that can be described as traumatic. Participants seem to adjust in ways that vary in the degree of constructiveness with regard to their well-being. They seem to go through a process of adjustment, reflecting psychological responses similar to the grief cycle model of Kübler-Ross (1969), Fisher's (2012) process of personal transition, and the stages in the transition curve described by Parker and Lewis (1981). During the initial stages of becoming a slaughterer and adjusting to the slaughterfloor context, they experience shock, similar to the immobilisation stage of Parker and Lewis (1981), as well as pertinently negative affective responses such as anxiety, fear, and guilt (compare transition process of Fisher, 2012). How well they cope with the traumatic effects of slaughter work during the following adjustment phase is dependant mostly on their perception of the work and the support they experience (Parker & Lewis, 1981) as well as their sense of coherence (Antonovsky, 1987). In this stage, some participants with a more positive outlook seem to be able to comprehend their work situation and find meaning and purpose in it (Antonovsky, 1987). Finding meaning enables them to engage in constructive coping tactics. Participants who seem less positive, reflect psychological defences such as denial (see Kübler-Ross, 1969; Parker & Lewis, 1981) and emotional detachment. When incorporating psychological defences in their coping strategy, participants seem to remain “stuck” in their struggle to move from languishing to flourishing (Keyes, 2002), consequently experiencing consistent negative work to home spill-over as well as social detachment and isolation.

## Reflections

The aim of this study was to develop a critical understanding of the well-being of employees working on the slaughterfloor of a commercial slaughterhouse in South Africa. Maladjustment in the workplace reflects behaviour that deviates from positive mental health and psychological well-being. This condition causes psychological discomfort and personal distress (Lowman, 1993; Nevid, Rathus, & Greene, 2008), as well as work-life conflict, deteriorated performance, workplace accidents, and absenteeism (Els & De La Rey, 2006; Hardy, Woods, & Wall, 2003). As is the case in our study, various instances of maladjustment have been reported in slaughterhouse studies. These include emotional detachment or “blunting,” substance abuse, concentration loss, sleep disturbances, depression, and destructive social consequences such as substance abuse and violence (Daly & Morton, 2008; Dillard, 2008; Henry, 2004; Hillier, et al., 2005; Human Rights Watch, 2004; Pearson, 2004).

Despite the mentioned psycho-social issues, people continue to work in slaughterhouses, seeing that these organisations play an important role in addressing various basic human needs. From the data, we developed an understanding of a slaughter worker's well-being from a process perspective, whereby slaughterers’ psychological responses and coping experiences manifest throughout the phases of becoming a slaughterer as well as adjusting to and maintaining their slaughtering work role. Our interpretations were primarily influenced by change models and theories related to responding to trauma (Kübler-Ross, 1969) and personal transition (Fisher, 2012) which reflected some of the symptoms we saw in the data such as denial, fear, anger, guilt, and depressive mood states. These symptoms were not only evident in participants’ narrated work-life experiences but also in the subconscious emotions and defences that were prevalent in the dreams they recalled. Work-related dreams or nightmares are not unusual since work may provide content for subconscious projections and processing of emotions related to work (Hill, 1996). The dreams narrated by slaughter workers were noteworthy in this study, especially because dreams often reflect a person's physical and mental well-being (Lowis, 2010). Our understanding of participants’ well-being as manifested in their work adjustment process was enhanced by the transition curve described by Parker and Lewis (1981) because participants demonstrated both destructive (maladjustment) and constructive adjustment. The transition curve allows for evidence of positive adjustment, resulting in an acceptance of reality, to an even more advanced stage where meaning is integrated in working towards continued constructive coping behaviours. The data spoke of how slaughterers employ psychological defences and presented with destructive coping tactics such as substance abuse as well as of finding strength and meaning in what they do to the benefit of sustaining their families and society.

Our interpretation of the data was furthermore guided by our meta-theoretical pre-understanding of well-being and mental health. Well-being has been defined as a broad state of physical, mental, and social health that includes general life satisfaction and the experience of more positive than negative affect (Koen, Van Eeden, & Rothmann, 2012). Keyes’ (2002, 2005) model of optimal mental health incorporates all aspects of well-being (Koen et al., 2012) and postulates that complete mental health is evident when there is (i) no diagnosable mental illness and (ii) flourishing (as opposed to languishing) is present. As such, people may function more towards the languishing pole of the flourishing–languishing continuum, without a psychological disorder necessarily being diagnosed (Keyes, 2002, 2005). Our data reflected psychological responses ranging on the spectrum of the flourishing–languishing continuum through the stages of adjusting to slaughter work. Most of the participants recounted their first slaughtering experience as traumatic, after which they admitted that they adjusted to the work setting by processing the experience of various negative emotions. As a result, unique psychological defences evolved and the employees applied behavioural coping mechanisms which though not always constructive, helped them maintain their ability to continue the work. Ultimately, participants showed evidence of languishing throughout the stages of becoming, adjusting to, and coping with slaughter work. Although in the coping phase participants also portrayed psychological responses reflective of moderate mental health and well-being, aspects of flourishing seemed limited in the data. Participants who were able to comprehend their life-work situation as understandable and meaningful (Antonovsky, 1987) move towards improved well-being when applying the bipolar health-ease versus dis-ease continuum (Strumpfer, Hardy, De Villiers, & Rigby, 2009). However, when applying the unipolar diagnostic perspective of Keyes (2005) in which pure mental health and pure mental illness is placed on one continuum and flourishing–languishing on the other, slaughterers do not consistently display adequate diagnostic criteria in lieu of flourishing. According to Keyes (2005), people who are moderately mentally healthy cannot be said to either flourish or languish in life and although moderately healthy people may function better than languishing people, they still function less effectively than flourishing or completely mentally healthy people. Reflected against Keyes’ (2005) categorical diagnostic of mental health, slaughterers’ negative emotional responses as well as their impaired functioning in terms of self-acceptance, social acceptance, relations with others, and social integration implies a lower subjective well-being and thus evidence of languishing.

Contemplating the mental illness–mental health continuum (Keyes, 2005) when considering the well-being of slaughterers, our understanding of diagnosable mental illness, and specifically the symptomology of post-traumatic stress syndrome (PTSD), impacted our interpretations. In the process of establishing themselves as slaughterers, participants to this study seemingly ran the risk of developing symptoms of PTSD, which is classified as an anxiety disorder with prolonged characteristic symptoms that develop after directly experiencing a traumatic event (Nevid et al., 2008). This syndrome provides a useful framework to understand and work with slaughterers on the issue of their well-being throughout the process of becoming and being a slaughterfloor employee. Dillard (2008) similarly identifies symptoms of PTSD in slaughterhouse employees caused by the consistent exposure to trauma in this work context and coined the term that describes the condition of “Perpetrator-Induced Traumatic Stress” (PITS) syndrome. According to various evolving perspectives, the diagnostic symptoms of PTSD mostly comprise three main symptoms, namely persistent re-experiencing, psychological and behavioural avoidance, and heightened arousal (Balduccia, Fraccaroli, & Schaufeli, 2011). In the fifth edition of the Diagnostic and Statistical Manual (DSM) of mental disorders, a fourth dimension was added: negative alterations in cognition and mood (Armour, 2015).

In the context of the study, evidence of participants who re-experience the trauma can be traced to the recurring nightmares and dreams they reported. All participants attested to suffering frequent bad dreams during the initial phases of employment. Even though less frequent, many continued to experience work-related nightmares, which reflected subconscious fear, shame, and guilt, as a result of their extreme work. Typical psychological defences such as emotional detachment, “doubling,” and aggressive behaviour manifested towards the self and others. For our research, these patterns demonstrate examples of psychological and behavioural avoidance. The confirmed instances of substance abuse can also be regarded as a form of avoidance behaviour. Various examples of heightened arousal can furthermore be isolated from the thematic results in the study, for example, sleep disturbances and sudden, unprovoked violent behaviour. With regard to negative alterations in cognitions and mood, the ultimate state of learned helplessness that results when employees accept the predicament of living with the psycho-social consequences of slaughterwork may be a case in point.

Our understanding of the initial trauma of having to slaughter, recurring nightmares that follow in the immediate period after employment, evidence of maladjustment and poor coping, as well as living with the psycho-social consequences of slaughtering work lead to an examination of the reasons for continuing to work on the slaughterfloor. This examination revealed a perception of limited resources and minimal alternatives for these employees. Seemingly they mostly feel forced to do slaughterwork and confess an inability to escape the role as they regard it as their sole competence and only means to provide for their families. Destructive behaviour towards others spill-over from the work to home environment. Such violence and participant experiences of social detachment and isolation intensifies negative affect and psychological defences that occurred earlier in the adjustment process, exacerbating in turn the negative spill-over and social consequences. If slaughter workers are unable to find meaning and comprehension, they ultimately begin to display a learned helplessness and an inability to reflect positively on their occupational choices and alternatives.

## Methodological considerations and recommendations

Due to the small contextual sample applied in this hermeneutic phenomenological study, the results cannot be generalised to the meat-processing industry as a whole. Moreover, the interpretation offered here has been impacted on by the specific meta-theoretical preconceptions of the researchers and offer only one view of the data. As researchers, we became cognitively and affectively involved in the data. We found it difficult to omit verbatim text as it was not easy for us to detach ourselves from the data. The reflections section in which we present our interpretive understanding of the data from meta-theoretical perspectives assisted us to gain a critical perspective. The internal consistency of our interpretation throughout the various analytical phases (Lindseth & Norberg, 2004) as well as the meta-theoretical reflections contributes to the trustworthiness of our approach. Multiple meanings and interpretations of the data are possible (Lindseth & Norberg, 2004) and we regard our interpretation as one alternative not excluding other possible interpretations. We believe that this study provides significant understanding of the uniquely challenging effect of slaughtering work on employees’ well-being and how well-being fluctuates on the flourishing–languishing continuum throughout the psychological adjustment process of being a slaughter worker.

In light of our reflections above, certain recommendations are proposed. Interventions aimed at addressing the well-being of slaughterfloor employees should be directed contextually. In other words, it should first be adapted to each phase of employment, targeting in succession: the initial phase of becoming a slaughterer, the adjustment phase, and the coping-and-maintenance phase. Second, interventions should address the psycho-social consequences from an individual and societal perspective. Regarding the focus on employee assistance, the PTSD framework can be used to diagnose psycho-social maladies and facilitate counselling and therapeutic interventions to deal with the symptoms that slaughterfloor employees experience as a result of their work.

Further research is needed to extend people's understanding of the PTSD syndrome underlying the unique experiences surrounding slaughtering for a living. Research should not only include other abattoirs but also focus on broader perspectives involving cultural differences with regard to slaughtering. The human resources function in the meat industry should renew its focus on well-being interventions and the psycho-social development of employees. This should apply not only to employees in the work context but also to the broader communities in which the abattoir is situated and in which the employees function, because social support and the development of internal psychological resources seems imperative to well-being in this context.
